# Genotypes, Antibiotic Resistance, and ST-8 Genetic Clone in *Campylobacter* Isolates from Sheep and Goats in Grenada

**DOI:** 10.1155/2014/212864

**Published:** 2014-02-11

**Authors:** Diana M. Stone, Yogesh Chander, Aschalew Z. Bekele, Sagar M. Goyal, Harry Hariharan, Keshaw Tiwari, Alfred Chikweto, Ravindra Sharma

**Affiliations:** ^1^School of Veterinary Medicine, St. George's University, P.O. Box 7, St. George, Grenada; ^2^Department of Veterinary Population Medicine, College of Veterinary Medicine, University of Minnesota, 1333 Gortner Avenue, St. Paul, MN 55108, USA

## Abstract

Rectal swabs from 155 sheep and 252 goats from Grenada were evaluated to determine the prevalence of *Campylobacter* spp., antibiotic resistance, and multilocus sequence types. Fifteen *Campylobacter* isolates were obtained (14 *C. jejuni* and 1 *C. coli*). The prevalence (3.7%) did not differ significantly between sheep (4.5%) and goats (3.2%). Among the seven antimicrobials tested, resistance was only detected for tetracycline (30.8%) and metronidazole (38.5%). *Campylobacter* isolates showed no significant difference between sheep and goats for type of antimicrobial resistance or percent of resistant isolates. Twelve of the isolates were successfully genotyped consisting of four recognized clonal complexes and three novel sequence types. Importantly, one isolate from one goat was identified as the *C. jejuni* sequence type-8, a zoonotic and tetracycline-resistant clone reported to be a highly virulent clone associated with ovine abortion in the USA. Although most samples were from comingled sheep and goat production units, there were no shared sequence types between these two host species. None of the sequence types identified in this study have previously been reported in poultry in Grenada, suggesting sheep- and goat-specific *Campylobacter* clones in Grenada. This is the first report of genotyping of *Campylobacter* isolates from sheep and goats in the Eastern Caribbean.

## 1. Introduction

Since its initial recognition in the 1970s, *Campylobacter* has become one of the most common causative agents of bacterial foodborne gastroenteritis in humans [1, 2]. The incidence of human *Campylobacter* infections is increasing worldwide along with the proportion of isolates resistant to antibiotics [3]. Animals, including sheep and goats, are the natural reservoir hosts for *Campylobacter *spp. Although most infections in small ruminants are asymptomatic, there is recent recognition of a zoonotic clone, sequence type- (ST)-8, which is associated with ovine abortions in a number of states within the United States [4, 5]. Recent studies report a wide range (5% to 49%) of *Campylobacter* prevalence in healthy sheep and goats from different geographic areas [6–9]. Several studies conducted in high sheep and goat producing areas indicate that these small ruminants may be important sources of human exposure [3, 6–15].

Grenada is a small, developing island state in the Caribbean where many residents are involved in small-scale sheep and goat production for meat, milk, and milk products. It is likely that, in a predominantly rural country like Grenada, sheep and goats serve as important sources for human exposure by direct contact or via environmental or food exposure. The goal of this study was to determine whether sheep and goats are a reservoir host for pathogenic and antibiotic-resistant *Campylobacter *spp. Another goal was to characterize *Campylobacter* population structure among these animals by multilocus sequence type (MLST) genotyping of the isolates.

## 2. Materials and Methods

### 2.1. Study Population and Sample Collection

Rectal swabs from 155 sheep and 252 goats from the islands of Grenada and Carriacou, islands in the tri-island state of Grenada, were obtained between May and July, 2011. The much smaller island of Carriacou is similar in land mass and population to the individual parishes in Grenada [16] and thus, for the purposes of this study, the island of Carriacou was considered as an island consisting of one parish. Based on the Grenada Ministry of Agriculture, Forestry and Fisheries report on the number of sheep and goats inspected in Grenada [17], we estimated a population size of 3000 sheep and 2500 goats on these 2 islands at the time of our sampling. Given these population estimates we sampled greater than 5% of the sheep and greater than 10% of the goats on these two islands. At the 95% confidence level our sample sizes were sufficient to detect a 5% fecal shedding rate with confidence intervals of ±3.34% and ±2.55% in sheep and goats, respectively (http://www.surveysystem.com/sscalc.htm).

Groups of sheep and/or goats owned by one person or family were defined as a production unit. Twenty-one production units were selected from the 6 parishes of Grenada and 8 production units were selected from the sister island of Carriacou. Production units ranged in size from 10 to 120 animals. Approximately 25% of the sheep and goats within these production units were sampled. Samples were from clinically healthy animals that were at least 6 months old. Health status was determined by physical exam by a veterinarian. Culture swabs containing Cary-Blair transport medium (BBL, Becton, Dickinson and Company, Sparks, MD, USA) were used for sample collection. Samples were transported on ice to the St George's University School of Veterinary Medicine Diagnostic and Research Laboratory for processing the same day.

### 2.2. Bacterial Culture and Identification

Rectal swabs were plated onto *Campylobacter* blood-free selective agar with cefoperazone and amphotericin B supplements (Modified CCDA-Preston plates, Oxoid Ltd., Basingstoke, UK). The plates were incubated under microaerophilic conditions at 42°C for 48 h using a microair atmosphere generating system (GENbox microaer system, 96126 BioMerieux UK Ltd, Basingstoke UK). Presumptive *Campylobacter *colonies were identified by their typical grey-white, mucoid flat appearance. *Campylobacter* morphology was confirmed by Gram staining. Growths of pure cultures were transferred into 2% sterile skim milk in cryovials and stored at −85°C. To speciate the isolates, the hippurate test was used as previously described [18]. A multiplex PCR that differentiates between *C. jejuni, C. coli, C. lari*, and *C. upsaliensis* on the basis of amplicon size of the *IpxA* gene was also used [19].

### 2.3. Antimicrobial Susceptibility Testing

Antimicrobial susceptibility/resistance of *Campylobacter* isolates (*n* = 15) was determined for the following seven antibiotics: gentamicin, chloramphenicol, ciprofloxacin, ampicillin, tetracycline, erythromycin, and metronidazole. Susceptibility and resistance to metronidazole has been proposed as an epidemiological marker for *C. jejuni* of avian origin [20] and may have relevance to ruminant-associated *C. jejuni* as well. The other six antibiotics are all used to treat human cases of campylobacteriosis. The Epsilometer test (*E-*test, AB Biodisk, Solna, Sweden) for minimum inhibitory concentration (MIC) was conducted according to the manufacturer's instructions on Mueller Hinton agar (Remel, Lennexa, KS, USA) as previously described [18]. A *C. jejuni* strain of poultry origin susceptible to all seven drugs and giving reproducible MICs was used as a control. The MIC of a drug was read directly from the scale printed on the *E*-test strip at the point of intersection between the zone of bacterial growth and the rest of the strip. Break points used by Guévremont et al. [21] and those established by the Clinical and Laboratory Standards Institute [22] for the microbroth dilution test on aerobic bacteria were used to interpret MICs. The break point for resistance to metronidazole was set at ≥16 *μ*g mL^−1^, as recommended by Lorian [23]. MIC values used to classify a strain as resistant were ampicillin ≥32 *μ*g mL^−1^, chloramphenicol ≥32 *μ*g mL^−1^, ciprofloxacin ≥4 *μ*g mL^−1^, erythromycin ≥8 *μ*g mL^−1^, gentamicin ≥16 *μ*g mL^−1^, tetracycline ≥16 *μ*g mL^−1^, and metronidazole ≥16 *μ*g mL^−1^.

### 2.4. Multilocus Sequence Typing (MLST)

Stock cultures of the 7 isolates from sheep and 8 isolates from goats were grown on blood agar plates for 24 h at 42°C under microaerophilic conditions. Total DNA was extracted using DNA blood and tissue purification kits (DNeasy Blood & Tissue Kits, Qiagen Inc., Valencia, CA, USA) according to manufacturer's instructions and samples were shipped to the College of Veterinary Medicine, University of Minnesota, St. Paul, MN, for MLST determination. PCR amplification of seven genes was carried out as described previously [24–26] using guidelines and PCR primer sequences obtained from the *Campylobacter *PubMLST database (http://pubmlst.org/campylobacter/mlst-info/Cjejuni/primers.html). Briefly, PCR was performed using commercial ready-to-use master mixes in a master cycler (Mastercycler (Eppendorf AG), HotStarTaq Master Mix kit, Qiagen Inc., Valencia, CA, USA). The reaction mixture contained 10 *μ*M of each primer, 25 *μ*L of PCR master mix, and 2 *μ*L of template DNA and water to make total volume of 50 *μ*L. The cycling program consisted of an initial denaturation at 95°C for 15 min, followed by 35 cycles of 94°C for 30 sec, respective annealing temperature for each primer for 1 min, and extension at 72°C for 3 min with a final extension at 72°C for 10 min. To confirm the presence of amplicon of the expected size, the PCR amplification products were run on 1.2% agarose gel. Purified PCR products were quantified and sequenced (ACGT, Inc., Wheeling, IL, USA) using the same primers used for the PCR amplification. The obtained sequences were analyzed using sequence analysis software (Sequencherver 10.1, Gene Codes Corporation, MI, USA). Sequence data were submitted to the *Campylobacter* PubMLST database for allele assignments.

### 2.5. Data Analysis

A two-tailed Fischer's Exact test was used to compare the percent of sheep and goats positive for fecal *Campylobacter*, prevalence rates among parishes, and the percent of isolates resistant to the various antibiotics. A value of *P* ≤ 0.05 was considered significant. Sequence analysis and determination of sequence types (ST) and clonal complex (CC) were performed with the integrated network MLST application for *Campylobacter *(SmartGene Inc, Raleigh, North Carolina, USA) which uses the PubMLST database http://pubmlst.org/campylobacter/ for ST and CC designation. A neighbor-joining tree based on allelic sequences was constructed using a suite of analysis programs (PHYLIP analysis programs) available on the PubMLST website http://pubmlst.org/analysis/. Tree drawing was performed using PubMLST Phylodendron software also available at the same PubMLST website. Tree branches are labeled by animal source (sheep or goat) and the corresponding ST assignment. Isolates not yet assigned to an ST are labeled as “Novel.”

## 3. Results

### 3.1. Production Unit Information

Of the 21 production units sampled in Grenada, 15 consisted of both sheep and goats and 6 consisted only of goats. None of the production units consisted only of sheep. Managers of sheep and goat production units on both islands reported no antibiotic use in their flocks.

### 3.2. *Campylobacter* Species

Fifteen *Campylobacter* spp. isolates were obtained from the 407 animals sampled (Table 1). All isolates were phenotypically typed by the hippurate test as either *C. jejuni* or *C. coli*, with 14 of the 15 isolates (93.3%) identified as *C. jejuni* (6 from sheep and 8 from goats) and 1 (6.7%) of the isolates identified as *C. coli* (from a sheep on the island of Carriacou). Results of speciation were further confirmed by multiplex PCR. The overall prevalence of *Campylobacter* fecal shedding was 3.7% and did not differ significantly between sheep (4.5%) and goats (3.2%) (*P* = 0.59). The sheep/goat prevalence ranged from 0% in the parish of St. George to 20% in the parish of St. Patrick. These were the only two parishes that differed significantly from each other for percent positive animals (*P* = 0.0019).

### 3.3. MLST Profiles and Antibiotic Resistance

MLST genotyping was completed on 13 of the 15 isolates (Table 2). For the other 2 isolates neither CC nor ST determinations were possible because amplification products were not detected for one or more loci. Although PCR conditions were optimized and sequencing was repeated, additional primers were not used to fill the missing gaps for ST designation due to limitation of sample. Five CCs identified in this study have previously been described (ST-21, ST-52, ST-353, ST-61, and ST-677). In addition, three novel clones not yet assigned to an ST were detected, one from a sheep and two from goats. Two CCs, ST-21 and ST-52, were identified in both sheep and goats. Within the CC ST-21, clones ST-50 and ST-454 were isolated from sheep and clones ST-8 and ST-5340 were isolated from goats. The two isolates from ST-52 were both novel. The only ST clone isolated from more than one animal was ST-50 which was identified in samples from 3 sheep. Among the *C. jejuni* isolates, five CCs were identified. The most frequent *C. jejuni* CC was ST-21 containing 7 of the 14 *C. jejuni* isolates.

Eight of the 15 *Campylobacter* isolates were resistant to either metronidazole or tetracycline or both (Table 3). There was no significant difference between the percent of isolates resistant to tetracycline (30.8%) and the percent resistant to metronidazole (38.5%) (*P* = 1.0). All of the metronidazole-resistant isolates were from Carriacou. This included 3 of the goat *C. jejuni* isolates and both of the *C. coli* isolates from sheep. Tetracycline-resistant isolates were from Carriacou, St. Patrick, and St. David and included the *C. coli* metronidazole-resistant Carriacou sheep isolate. All four of the tetracycline resistant isolates belonged to the ST-21 clonal complex and encompassed 3 of the 4 ST-21 clones.

**Figure 1:**
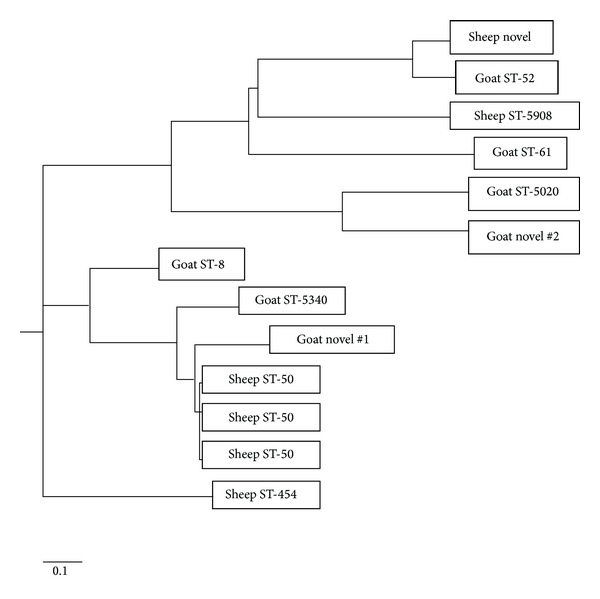
Neighbor-joining tree constructed from 8 ST clones and 3 novel clones identified among 13 *Campylobacter jejuni* isolates from Grenadian sheep and goat fecal samples. Tree branches are labeled with the corresponding ST assignments.

A cladogram constructed from the 13 typable *Campylobacter* MLST allelic profiles illustrates the clustering of the STs identified in this study and demonstrates the genetic relatedness of sheep and goat isolates and the lack of any shared STs between sheep and goats (Figure 1).

## 4. Discussion

The aims of this study were to determine the prevalence, genetic diversity, and antibiotic resistance of *Campylobacter* isolates from sheep and goats in Grenada. The *Campylobacter* prevalence in sheep (4.5%) and goats (3.2%) in this study is generally lower than that reported from other geographic areas [6, 7, 9, 12, 15]. However, similar to these reports and the PubMLST data base, the majority of our isolates were *C. jejuni. C. coli* was isolated from only one sheep from the island of Carriacou. Because this isolate was not MLST typable, one must consider the possibility that the isolate was not a *Campylobacter.* The low prevalence results from our study may reflect sample size, sampling biases, or the lack of *Campylobacter* MLSTs in Grenada that predominantly colonize sheep/goats. The lower prevalence noted in this study may also be due to the fact that the animals in Grenada are kept in a free range system and there is no intensive farming on the island. Enrichment methods were not used because, compared to direct plating, they were shown to be inefficient for isolation of *Campylobacters* from fecal samples from animals [27–29]. Musgrove et al. [30] noted that large numbers of non-*Campylobacter* species that inhabit the intestinal tract may outcompete *Campylobacters* during enrichment, confounding detection. However, information on enrichment techniques with regard to fecal sample of sheep and goats is lacking, and further research may elucidate this aspect. Most of the ST clones identified in our study are STs that are globally associated with poultry as a host species (*Campylobacter* PubMLST Database, 2012). Regardless, *C. jejuni* and possibly *C. coli* are present in small ruminants in Grenada.

In this study we identified eleven ST clones including eight previously reported STs within five recognized CCs and three novel clones not yet assigned. Of major interest is the detection of the previously reported clone ST-8 in CC ST-21 in a single goat in our study. It was recently reported that the vast majority of *C. jejuni* isolates associated with sheep abortion in several US states belonged to this single ST-8 clone, referred to as clone SA for sheep abortion. Further, as with the isolate from the goat in this study, all were resistant to tetracycline, the only antibiotic class currently approved in the USA for treatment of *Campylobacter* abortion in sheep [4]. More recently clone SA was reported in association with human disease [5]. Although the PubMLST database only shows one report of ST-8 in a goat, other goat ST-8 isolates have been reported in association with abortions [5]. Because not all ST-8 isolates may be the SA clone, additional testing of our ST-8 isolate would be required to determine if it is indeed identical to the SA clone. Because sheep or goat abortion cases in Grenada are rarely seen by a veterinarian and never diagnostically evaluated, there is no information on *Campylobacter*-associated ovine or caprine abortions on the island. Unlike in the USA, antibiotics are not used in small ruminant production in Grenada and thus the detection of the tetracycline resistant ST-8 clone on the island cannot be explained by tetracycline use in these animals. It is possible that importation of sheep and/or goats into Grenada accounts for clone ST-8 being present on the island. Of interest is that documented importation of sheep into Grenada during the last 20 years has only occurred from the island of Barbados and prior to 2004. However, there is one documented shipment of goats into Grenada in 2007 from the United States (B. Louison, Grenada Ministry of Agriculture, Forestry and Fisheries, personal communication, 2011). Undocumented importations may also have occurred.

The major lineages identified in this study correspond to CCs that are primarily associated with sporadic human disease and poultry and are geographically widely distributed (Table 4). However, none of the sheep/goat isolates from this study correspond to any of the STs previously identified in poultry in Grenada [18]. Because of the type of agriculture practiced in Grenada where biosecurity between various animal production units is minimal and comingling of species is common, these observations support host species-associated *Campylobacter* STs due to factors other than host species containment. This observation is similar to results from a study in Scotland of sheep and cattle exposed to the same farming environment, but that consistently showed significant differences in their carriage of *Campylobacter* species, STs, and CCs [31].

The *Campylobacter* isolates (*n* = 15) genotyped into 11 different STs, indicating considerable population diversity in both sheep and goats. Four of the 8 previously reported STs identified in this study (ST-21 sequence type 454, ST-21 sequence type 5340, ST-677 sequence type 5020, and ST-353 sequent type 5908) have never been reported from sheep before. Five of the 8 previously reported STs identified in this study have never been reported in goats before, with only ST-21 sequence type 50 and sequence type 8 being previously reported. Most of our isolates represent STs reported from the UK, The Netherlands, Germany, and Canada (Table 4). Two of the STs from our study have previously been reported from the Caribbean island of Curacao, all 12 of which were from human cases of gastroenteritis (*Campylobacter* PubMLST Database, 2012).

CC ST-21 was the dominant CC for both sheep and goat isolates (Table 2) and is represented globally and from multiple host species including sheep and goats (Table 4). Within this complex, clone ST-50 accounted for 3 of the 4 sheep isolates. CC ST-21 was also the only shared complex between sheep and goats and the only complex with multiple isolates from both host species. At the ST level, however, there were no shared clones between sheep and goats. Because the majority of our samples were from sheep and goat comingled production units, these results further support the existence of different sheep-associated and goat-associated *Campylobacter* clones. Currently the PubMLST database has only 9 *Campylobacter* submissions from goats and 162 from sheep (*Campylobacter* PubMLST Database, 2012). Thus, it is not yet possible to know whether different sheep- and goat-associated *Campylobacter* STs exist globally. Determining the clonal population structure for *Campylobacter* among small ruminants is of interest in terms of potential links to antibiotic resistance and transmission to humans.

The *Campylobacter* isolates showed no significant difference between sheep and goats for either the type of antibiotic resistance or the percent of antibiotic resistant isolates (Table 3). Resistance was only detected for tetracycline and metronidazole. The level of *Campylobacter* tetracycline resistance in this study (57% of ovine isolates and 50% of caprine isolates) is in close agreement with those from Canada [32], Spain [33], and Iran [8, 12]. The *E*-test results are known to correlate well with agar dilution and broth dilution methods for erythromycin and ciprofloxacin. However, it has been noted that the overall agreement between *E*-test and broth microdilution method is only 90%, and even less with agar dilution methods [34, 35].

Results from this study contribute to the understanding of *Campylobacter* population structure within and among host species in Grenada. Sheep and goat rearing in this island state consists of small production units which are often in close association with back yard or small-scale poultry and swine production. This type of animal agriculture is common in many parts of the world and findings from Grenada may shed light on *Campylobacter* population structure, spread, and antimicrobial resistance relevant to many geographic areas.

## 5. Conclusions

This report of genetic typing of *Campylobacter* isolates from sheep and goats is the first in the Eastern Caribbean. Results from this study document that both sheep and goats are reservoirs for antibiotic resistant *Campylobacter jejuni/coli*. Results also document that the previously identified, highly pathogenic *Campylobacter jejuni* genetic clone for ovine abortion in the USA, ST-8, is also present in Grenada. Identical to the US isolates, the ST-8 isolate from a goat in Grenada is also tetracycline-resistant. *Campylobacter* isolates in this study are primarily from comingled sheep and goat production units and yet no shared genetic clones were identified between these two host species. None of the sequence types identified in this study match those previously reported in poultry in Grenada, suggesting the presence of sheep- and goat-specific *Campylobacter *clones in Grenada.

## Figures and Tables

**Table 1:** The prevalence of *Campylobacter *in sheep and goats from the six parishes of Grenada and from Carriacou.

Parish	Number of sheep/goat sampled	Number (%) of *Campylobacter* in sheep		Number (%) of *Campylobacter* in goats		Total number (%) of *Campylobacter *isolates
		*C. jejuni *	*C. coli *	*C. jejuni *	*C. coli *	
St. George	4/66	0 (0.0)	0 (0.0)	0 (0.0)	0 (0.0)	0 (0.0)
St. David	31/60	0 (0.0)	0 (0.0)	2 (3.3)	0 (0.0)	2 (2.2)
St. Andrew	23/47	0 (0.0)	0 (0.0)	2 (3.3)	0 (0.0)	2 (2.9)
St. Mark	6/14	1 (16.7)	0 (0.0)	0 (0.0)	0 (0.0)	1 (5.0)
St. Patrick	19/1	4 (21.0)	0 (0.0)	0 (0.0)	0 (0.0)	4 (20.0)
St. John	18/29	0 (0.0)	0 (0.0)	1 (3.4)	0 (0.0)	1 (2.1)
Carriacou	54/35	0 (0.0)	2 (3.7)	3 (8.6)	0 (0.0)	5 (5.6)
Total	**155/252**	**5(3.2)**	**2 (1.3)**	**8 (3.1)**	**0 (0.0)**	**15 (3.7)**

**Table 2:** Clonal complexes and allelic profiles from 15 *Campylobacter jejuni/coli* strains isolated from sheep and goats in Grenada and Carriacou.

Host	CC^a^	ST^b^	Allele number						
			ASP	GLN	GLT	GLY	PGM	TKT	UNC
Sheep	ST-21	50 (3)^c^	2	1	12	3	2	1	5
		454 (1)	2	1	1	5	22	1	8
	ST-52	**#1 Novel **(1)^d^	9	25	2	10	104	3	6
	ST-353	5908 (1)	7	2	5	2	104	3	6
	Untypable sheep	? (1)	Close 28	4	12	Close 28	Close 1	?	5

Goats	ST-21	8 (1)	2	1	1	3	2	1	6
		5340 (1)	2	1	12	3	104	1	5
	ST-52	52 (1)	9	25	2	10	22	3	6
	ST-61	**61 (1)**	1	4	2	2	6	3	17
	ST-677	**5020 (1)**	10	2	50	62	120	76	52
	ST-21	**#1 Novel **(1)^d^	2	39	12	3	2	1	5
	ST-677	**#2 Novel** (1)^d^	33	39	50	Close 47^e^	120	76	52
	Untypable goat	? (1)	Close 28	?	Close 2	Close 28	Close 1	?	6

^a^Clonal complex.

^b^Sequence types.

^c^Numbers in parentheses after each ST denote the number of isolates.

^d^No exact matches yet in the PubMLST data base for ST determination.

^e^Allele 47 for GLY was used for CC determination.

**Table 3:** Antibiotic resistance of the 15 *Campylobacter jejuni/coli *isolates from sheep and goats in Grenada and Carriacou.

Antibiotic^a^	Sheep (7 isolates) Number of resistant isolates/%	Goats (8 isolates) Number of resistant isolates/%	Total (15 isolates) Number of resistant isolates/%
Tetracycline	3^b^/42.9%	1/12.5%	**4/30.8%**
Metronidazole	2^b^/28.5%	3/37.5%	**5/38.5%**

Total	**4** ^ b^ **/57.1%**	**4/50%**	**8** ^ b^ **/53.3%**

^a^Antibiotics tested but no resistance found: Ampicillin, Chloramphenicol, Ciprofloxacin, Erythromycin, Gentamicin.

^b^One sample was resistant to both tetracycline and metronidazole.

**Table 4:** *Campylobacter* Clonal complexes from sheep and goats in Grenada for source, country, and human epidemiology as reported in pubMLST (accessed November 21, 2012).

CC^a^	ST-21	ST-21	ST-21	ST-21	ST-61	ST-52	ST-677
ST^b^	50	454	8	5340	61	52	5020
(number of isolates)	(570)	(2)	(40)	(1)	(282)	(95)	(1)
(%*C.j/*%*C.c*)	(100/0)	(100/0)	(100/0)	(100/0)	(98.58/1.42)	(98.95/1.05)	(100/0)
Source							
Human	77.03%	100%	50%	100%	62.41%	83.16%	100%
Poultry	16.92%		17.50%		3.19%	10.53%	
Cattle	2.59%		12.5%		24.12%	—	
Sheep	o.69%		12.5%		6.74%	5.26%	
Goat	0.17%		2.5%		—	—	
Environment	0.52%		5%		0.35%	—	
Country							
UK	48.42%		7.5%	100%	63.12%	67.37%	100%
Netherlands	17.37%		0		7.45%	9.47%	
Germany	8.42%		0		2.84%	—	
Canada	7.72%		37.5%		12.41%	12.63%	
Thailand	2.81%		0		—	—	
Japan	2.28%		7.5%		2.84%	1.05%	
USA	0	100%	47.5%		4.61%	—	
Curacao	2.11%		0		—	1.05%	
Human epidemiology							
Sporadic	43.33%	100%	22.0%	No value	32.98%	49.47%	No value
Outbreaks	0.18%		27.5%		1.06%	No value	No value

^a^Clonal complex.

^b^Sequence types.
